# Artificial intelligence with multi-functional machine learning platform development for better healthcare and precision medicine

**DOI:** 10.1093/database/baaa010

**Published:** 2020-03-17

**Authors:** Zeeshan Ahmed, Khalid Mohamed, Saman Zeeshan, XinQi Dong

**Affiliations:** 1 Institute for Health, Health Care Policy and Aging Research, Rutgers, The State University of New Jersey, 112 Paterson Street, New Brunswick, NJ, USA; 2 Department of Medicine, Rutgers Robert Wood Johnson Medical School, Rutgers Biomedical and Health Sciences, 125 Paterson Street, New Brunswick, NJ, USA; 3 Department of Genetics and Genome Sciences, School of Medicine, University of Connecticut Health Center, 263 Farmington Ave., Farmington, CT, USA; 4 Institute for Systems Genomics, University of Connecticut, 67 North Eagleville Road, Storrs, CT, USA; 5 The Jackson Laboratory for Genomic Medicine, 10 Discovery Drive, Farmington, CT, USA

## Abstract

Precision medicine is one of the recent and powerful developments in medical care, which has the potential to improve the traditional symptom-driven practice of medicine, allowing earlier interventions using advanced diagnostics and tailoring better and economically personalized treatments. Identifying the best pathway to personalized and population medicine involves the ability to analyze comprehensive patient information together with broader aspects to monitor and distinguish between sick and relatively healthy people, which will lead to a better understanding of biological indicators that can signal shifts in health. While the complexities of disease at the individual level have made it difficult to utilize healthcare information in clinical decision-making, some of the existing constraints have been greatly minimized by technological advancements. To implement effective precision medicine with enhanced ability to positively impact patient outcomes and provide real-time decision support, it is important to harness the power of electronic health records by integrating disparate data sources and discovering patient-specific patterns of disease progression. Useful analytic tools, technologies, databases, and approaches are required to augment networking and interoperability of clinical, laboratory and public health systems, as well as addressing ethical and social issues related to the privacy and protection of healthcare data with effective balance. Developing multifunctional machine learning platforms for clinical data extraction, aggregation, management and analysis can support clinicians by efficiently stratifying subjects to understand specific scenarios and optimize decision-making. Implementation of artificial intelligence in healthcare is a compelling vision that has the potential in leading to the significant improvements for achieving the goals of providing real-time, better personalized and population medicine at lower costs. In this study, we focused on analyzing and discussing various published artificial intelligence and machine learning solutions, approaches and perspectives, aiming to advance academic solutions in paving the way for a new data-centric era of discovery in healthcare.

## 1. Introduction

**Figure 1:**
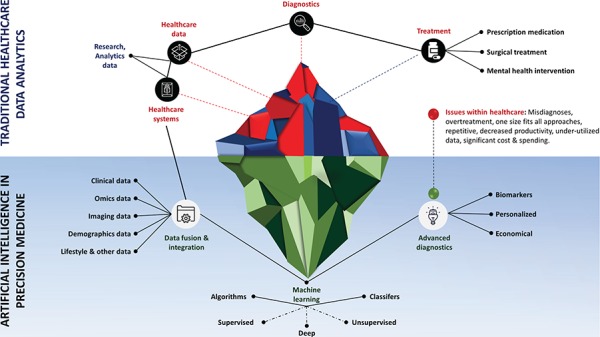
Role of artificial intelligence in traditional healthcare data analytics, and in precision medicine. Addressing key issues in healthcare (e.g. misdiagnoses, overtreatment, one-size-fits-all approaches, repetitive, decreased productivity, under-utilized data, significant cost & spending), and finding key biomarkers to provide economic and personalized treatment by intelligently analyzing heterogeneous data.

Over the centuries, quests for answers have led us to take giant leaps. It was only in the last century that the discovery of antibiotics freed us from many of the dreaded diseases of the past. Still, in the context of recent published literature (e.g. accessible through PubMed), over 138 000 studies discuss medication errors, and over 450 000 include delayed treatment (date accessed 10 October 2019, using search query keywords including ‘medication error’ and ‘delayed treatment’). Still, the problem of people dying from medical care gone wrong has been vastly underappreciated and not well recognized. Today, we stand on the threshold of the new medical revolution, just as big and far-reaching. Despite all of our scientific knowledge, much of medicine is still based on the treatment of symptoms and performing learned trials based on treatments, which works for most patients to bring symptom relief, reduce the risk of complications and improve survival chances, but not for all. To get new insights into disease taxonomy, etiology and pathogenesis, it is important to understand how diseases are related to each other. Breakthroughs in prescription medication, surgical treatment and mental health interventions are among the reasons we live longer. However, providing the correct in-time treatment plan for patients with knowledge about their current medications and drug allergies is currently a tedious and error-prone task [1]. The widespread growth of prescribing and consuming medications has increased the need for applications that support medication reconciliation. Furthermore, living healthcare issues include misdiagnosis, overtreatment, decreased productivity, under-utilized clinical data handling, significant cost and spending (Figure 1). These miscalculations can be reduced to a great extent with the use of advancements in information technology at every level of care.

Medical error is the third leading cause of death after heart failure and cancer (2). According to recent studies, approximately 180 000 to 251 000 people are dying every year in the USA due to medical errors (2). This number has been rising due to increasing complexity and reduced quality of our current medical system, which includes communication breakdown, misdiagnosis, poorly coordinated care and growing cost. In recent years, the concept of precision medicine has evolved as a central innovation pillar for leading research in transforming health and holds great promise in patient treatment (3,4). Precision medicine has the potential to improve the traditional symptom-driven practice of medicine by intelligently integrating multi-omics profiles with clinical, imaging, epidemiological and demographic details to allow a wide range of earlier interventions for advanced diagnostics and tailoring better and economical personalized treatment (Figure 1). This requires a progressive healthcare environment that can enable clinicians and researchers to gain a complete picture of the patient to deepen their understanding, using additional basic details from healthcare data e.g. phenotypic information, life style factors and social determinants that can impact treatment decisions. It is primarily based on ‘4Ps’—Predictive, Preventive, Personalized and Participatory—treatment of each individual patient and aims to enable clinicians to efficiently understand how personalized clinical data variations can contribute to health and accurately diagnose and predict the most appropriate course of action for a patient (5). While the complexities of diseases at the individual level have made it difficult to utilize healthcare information in clinical decision-making, some of the existing constraints have been minimized by technological advancements (6). To implement effective personalized and population health with enhanced ability to positively impact patient outcomes, it is important to harness the power of electronic health records (EHR) by integrating disparate data sources and discovering patient-specific patterns of disease progression to provide real-time decision support. The significance of healthcare data mining cannot be denied, but the challenges of big data management loom large (7).

Over the years, biotechnology has evolved immensely. Computers are becoming faster in speed and micro in size, heterogeneity is increasing in datasets and their volume is growing robustly. These expansions are fueling the engine of artificial intelligence (AI) for discovering many technical refinements to solve complex problems in almost every field of life, including science and medicine. AI is the branch of computer science with the capability of a machine to imitate and even enhance intelligent human behavior. One of the expected roles in life and medical sciences is to deal with extensive research studies aimed at supporting real-time decision-making and producing solutions to complex problems through knowledge and data intensive computational and simulated analysis (8). Healthcare data includes information about a patient’s lifestyle, medical history, encountered visits with practices, laboratory and imaging tests, diagnoses, prescribed medications, performed surgical procedures and consulted providers (9). Adequate, analytical and intelligent access to healthcare data has the potential to revolutionize the field of medicine by improving the quality and transition of care, improving outcomes by reducing cost, detecting diseases at earlier stages (10,11) and developing a better understanding of biological mechanisms by modeling complex biological interactions through a holistic integration and analysis of knowledge (12). The ability to stratify patients, understand scenarios and optimize decision-making would consistently improve based on the myriad data obtained during the care-delivery process. Innovative and robust big data platforms are necessary to improve the quality and transition of healthcare by analyzing heterogeneous healthcare, which can be of huge volume, velocity, variety and veracity.

To effectively implement healthcare data analytic processes, various big data management challenges (1,9,13,14,15,16,17,18,19) have to be overcome, which include inadequacy of analyzable clinical data (20); existence of multiple data standards, structures, types and formats; rapid growth in heterogeneous data; understanding of analysis algorithms for clinical data interpretation, exploration and drawing inference; unavailability of effective open-source tools that combine various approaches to model biological interactions; integration of clinical and analytic systems; interdisciplinary field barriers; high cost (21); implementation of secure frameworks for data collection, simplification, conversion from raw form to knowledge, management and distribution (22,23); automatic cleansing of faulty and error-prone EHRs; correctly identifying prescription medication; and implementing predictive diagnostics (10). It is not possible to easily track and prospectively follow the clinical progress and outcomes in patients over time (e.g. having a critical predictor of future clinical events where a patient may show the disease months or years down the road). In past decades, various systems have been developed in both commercial and academic sectors (11,24,25,26,27,28,29,30) for this purpose. Academic systems put significant values on analytics, while commercial systems focus on supporting clinical operations. However, independently utilizing traditional approaches, both sectors are unable to identify problems by their effects and significantly help in clinical decision-making. However, major concerns include handling and evaluation of electronic medical records (EMR), and repetitive tasks; patient medication adherence; inefficient therapeutic treatment for cancer and other critical diseases; cost ineffectiveness; and addressing ethical issues related to AI and ML implementation in healthcare.

## 2. AI & ML in health intelligence, precision medicine and resource management

Intelligent big data platforms are necessary to improve the quality and transition of healthcare by expediting investigation of active hidden factors in clinical data with machine learning algorithms to obtain actionable gap-based information about patients for early detection and prevention of constitutional disorders like cancer, and streamlining data sharing by developing efficient communication across healthcare units and scientific laboratories. Its application in healthcare could be another great leap in medicine and a transformational force for guiding personalized and population medicine with several computational benefits. In the recent past, multiple AI and ML-based efforts have been made for deciphering diseases to facilitate predictive diagnosis and thereby guide treatment factors, e.g. drawing disease relationships using clinical manifestations, EHR and data generated using wearable technology. To get a detailed overview of available academic solutions, we reviewed contributions and compared various AI and ML claimed solutions, approaches and discussions (Table 1), and real-time examples (Table 2) published within the last 5 years [31–63]. Our focus here was to discuss valued contributions of all mentioned AI and ML algorithms (section Theoretical background of AI, ML and examples in healthcare and approaches. We provide detailed and individualized overview of presented approaches. Overall, the review study and the contributions of AI and ML are divided into three categories: Health Intelligence, Precision Medicine, and Healthcare Resource Management and Ethical Challenges.

**Table 1:** Feature and variability analysis of reviewed approaches, and real time implementation of ML algorithms

**Approach**	**Objectives**	**Approach**	**AI & ML**
AI power digital medicine (31)	Reduce repetitive tasks and burdens of electronic medical records through the utilization of AI and ML.	Increased task automation with improved image processing. Monitor medication adherence and detect any changes.	Deep convoluted neural network for skin cancer detection and reducing visit length. Deep neural network to evaluate images for diabetic retinopathy. Smartphone-based AI platform to measure adherence in patients on direct oral anticoagulants.
ML in medicine (32)	Examining the essential structural changes in the healthcare system that are necessary to unleash the full potential of machine learning in medicine.	Accumulation of large data set and implement ML to anticipate events, develop search engine, and monitor data flow.	Applied deep learning on the current EHRs data to generate associations and meaningful data for personalized diagnosis and treatment.
Precision medicine with electronic medical records (33)	Applying ML to the EHRs to generate personalized medicine by converting EHR into reliable risk predictors, and incorporating patient’s variabilities for treatment and prevention of disease.	Analyze patterns within the subset of population who present similar clinical phenotypes of complex disease.	Supervised learning (support vector machine, discriminant analysis, naïve Bayes, nearest neighbor and neural network), unsupervised learning encompass (linear & logistical regression, decision tree, cluster analysis, and neural network).
AI, ML and the evolution of healthcare (34)	Examining AI integration in healthcare.	AI methods for the extraction of big data and aid clinicians in care delivery	SVM model development for physiological data segmentation and analysis, disease progression prediction, and diagnosis.
Solving healthcare problems with precision medicine (35)	Tailoring medical treatment with respect to the individualized characteristics of patients.	Use of information technology for multidisciplinary collaboration establishment between clinicians and researchers.	ML for the implementation of precision medicine, which includes data storage and analysis for determining the association between disease outcome (e.g. disease risk, prognosis, or treatment), identification of patient characteristics and optimal treatment.
Role of AI in precision medicine (36)	Examining role of AI in precision medicine implementation.	Analyzing large scaled clinical dataset.	Combining DL with human pathologist to improve success rate of diagnosis.
AI towards health in resource-poor settings (37)	Utilization of AI in poor settings, and improving health outcome in those areas.	Implementing NLP over EHR for surveillance and out breaking predictions.	Pattern identification and tracking disease transmission through ML.
Integrated precision medicine and role of EHR in personalized treatment (38)	Early diagnosis of chronic conditions through proper extraction of clinical insights.	Feature extraction from clinical data, and utilization of silico dataset.	Predictive, proactive intervention in healthcare through AI, and clinical decision support tool development.
AI in healthcare (39)	Analyzing AI applications in healthcare, and their potential outcome in future.	Precise analysis at the extracted useful information from a large patient population.	ML algorithms to extract and cluster data, and perform principal component analysis, SVM to determine model parameters, and identify imaging biomarkers, NLP for text processing and classification, and DL for diagnostic imaging and electro diagnosis.
ML Knowledge Base with ontology for pattern recognition in personalized medicine (40)	Examine three main pillars integrating personalized medicine into everyday clinical practice, which are phenotype categories, population size and statistical analysis.	Developing knowledgebase of existing phenotypes, patient enrollments, and data expansion.	ML approaches for pattern recognition and development of statistical models (sample size and effect size). Knowledge base of all existing phenotype categories and disease. Organized clinical dataset of population size. Software platform for statistical analysis of high-dimensional healthcare and multi-omics data.
Data science, AI, and ML for laboratory medicine (41)	Predictive modeling for better collaboration between hospitals without sharing data and complying privacy regulation.	Data Science (DS) and AI to mimics the human processes and improve the process of decision-making.	ML for healthcare data analysis and optimization, and reducing cost, improving efficiency of staff and resources.
AI to solve the human resource crisis in healthcare (42)	Solve the human resource crisis in healthcare with AI.	Implementation of AI techniques (43).	Artificial narrow intelligence for performing a single task. Artificial general intelligence for understanding and reasoning environment like humans. Artificial superintelligence for scientific creativity. Deep learning for image recognition, natural language processing and translation.
Data analytics and ML for disease identification in EHR (44)	Analyzing EHR for the identification of wide range of medical conditions and diagnosis.	Converting electronic healthcare record into reliable risk predictors.	ML algorithm for structured and unstructured big data analysis for the identification of wide range of medical conditions and diagnosis.
AI, Big Data and Cancer (45)	Application of AI and large scaled database for cancer diagnosis and treatment, worldwide.	Application of cognitive computer systems for approaching cancer diagnosis and treatment (read, remember, recommend, and remind).	Cognitive computer systems for providing rapid access to accurate information and treatment procedures, and assisting in decision-making.
Use of EHR in comparative effectiveness research (46)	Reporting caveats in existing healthcare systems.	Literature review and reporting caveats.	Implementing ML for overcoming existing big data limitations in healthcare systems.
Deep learning health care system (47)	Reporting unintended consequences due to the application of ML in existing healthcare systems.	Creating more precise analytics platform for prognosis modeling and pattern recognition.	ML for prognosis modeling in oncology, and pattern recognition in radiology and pathology.
DL to transform healthcare (48)	Transform healthcare by using ML.	Outperforming clinical systems and modeling complex relationships among active hidden factors of data	Implementation of DL for the digital image analysis.
High-performance medicine with AI (49)	Exploring importance and pitfalls of AI in medicine.	Literature review and field analysis.	Deep neural networks for pattern recognition and analysis medical images. NLP in drug discovery by analyzing biomedical literature.
Intelligent digital pathology (50)	Improving diagnostic accuracy and efficiency with the use of ML.	Implemented, examined and compared the performance of DL at test dataset.	DL for analyzing whole-slide pathology images.
ML for prediction in EHR (51)	Implementing ML for better understanding heterogeneous treatment effects to implement precision medicine.	Evaluating positives and negatives of ML algorithms.	ML algorithms for addressing different clinical questions by analyzing and finding nonlinear relationships in the EHR.
Unintended consequences of ML (52)	Safe, effective, efficient and humanistic care.	Implementing DL in healthcare analytic systems development and modeling tools.	DL for digital imaging, curating data sets, integrative heterogeneous data analysis, identifying novel associations, and remote monitoring and digital consultations.
Finding the missing link for big biomedical data (53)	Biomedical data integration and analysis located at heterogeneous sources.	Identify and discussed challenges in biomedical data linking.	AI and ML tools development to analyze biomedical data for better clinical decision-making.
ML classifies cancer (54)	Identification of novel tumor classes.	Application of ML for the identification of tumor by analyzing histology and genomics data.	ML for analyzing histological data. Supervised ML for analyzing CNS tumor type genome-wide methylation data to identify methylation patterns. Unsupervised ML to search patterns in the data sets to develop classification categories.
Analyzing and visualizing knowledge structures of health informatics (55)	Finding future strands of research, including new algorithms, tracking tools and Internet of Things-based decision support systems.	Quantitative review of the health informatics field, employing text mining and bibliometric research methods.	DL, new ML algorithms and advanced big data analytics for better-personalized treatment.
Big data and ML algorithms for healthcare delivery (56)	AI tools development based on incremental learning to refine the predictive accuracies.	Identification of clinical problems, annotation of extracted healthcare data, application of appropriate ML algorithms and its effect on decision-making, addressing legal and ethical implications, assessment of ML effect in trail, designing freeze and submission of dossier for medical devices, training clinicians of ML tool, and monitoring for adverse outcomes.	HCI-based AI and ML applications for different clinical developments in oncological.
Intelligent health data analytics (57)	AI for advanced health data analytics.	Health data analytics process involving a methodical order of data processing, modeling, and analysis steps.	Implementation of AI and ML based analysis with the inclusion of health data preprocessing, selecting algorithm based on expected outcome, developing analytical models, and interpreting results.
Ethical challenges of implementing ML in healthcare (58)	Challenges of implementing ML in healthcare	Literature review and field analysis.	Addressing current challenges in healthcare systems due to the implementation of ML.
Data science, AI, and ML for laboratory medicine (59)	Implementing Data science, AI, and ML for laboratory medicine.	Framework including defining tasks, metrics, models and datasets.	ML for finding patterns, discovering inefficiencies, predicting outcomes and taking factual decisions.
Causal inference and ML (60)	Examined the implications of progress in observational research design and healthcare databases.	RWE framework.	ML for data classification and prediction in RWE to support clinical and regulatory decision-making.
Big data analytics in healthcare (61)	Application of big data analytics in healthcare.	Conceptual architecture of big data analytics, which includes developing multi source data input, transformation, structure, management and analysis using traditional SQL, OLAP and mining.	ML for data mining and analysis.
ML and genomics in precision medicine (62)	Substantial improvements to address clinical and genomic data security problems.	Combining the latest computational data protection principles with legal and ethical perspectives to construct a secure framework for data sharing.	ML models to address the challenges of gene variations and similarities among patients.
ML in cancer prognosis and prediction (63)	ML to detect key features by predictive modeling of complex and heterogeneous datasets for progression and treatment of cancerous conditions, risks and outcomes.	Data preprocessing with focus on data modification via dimensionality reduction and feature detection.	ML (ANNs, BNs, SVMs, graph-based SSL and DT) to model the progression and treatment of cancerous conditions thru examining complex datasets and revealing their relevance.

## 3. Health intelligence approaches

Health intelligence can play a vital role at various levels of clinical research and analytics that can lead to significant improvements in achieving goals for the provision of better personalized and population healthcare. In the past decade, various operational and research-based healthcare data management and analytic systems have been developed in both academia and commercial sectors. Our interest includes comprehensive solutions that implement healthcare data analytics process; provide features to manage, analyze, visualize and share EHR in de-identified form; help in automatically capturing information about patient demographics, scheduled appointments, pre-exam questionnaire results, consulted providers, conducted lab tests, diagnoses, treatment plans, objective test results, medications, surgical procedures and claims; support the clinical decision-making process with AI techniques to create classifiers, which can be trained on structured clinical data generated from different clinical activities and can learn similar groups of subjects, associations between subject features and outcomes of interest; and apply natural language processing (NLP) methods to extract information from unstructured clinical data e.g. narrative text, such as physical examination, clinical laboratory reports, operative notes and discharge summaries.

### 3.1. AI in healthcare for better prevention, detection, diagnosis and treatment of disease (31)

The authors emphasized the idea of embracing changes with the advancement in technology with the potential integration of AI into the field of healthcare in a way that is beneficial to each healthcare worker. They focused in utilizing AI to obviate repetitive tasks to enhance patient–physician relationship and increase practice of empathy and emotional intelligence. Authors focused on deep learning algorithm implementation with increased data flow that allows machines to self-develop a complex function with improved predictability, as long as a large amount of data is fed as input. They developed a deep convoluted neural network for skin cancer detection, image analysis for diabetic retinopathy evaluation, smartphone-based AI platform to measure adherence in patients on direct oral anticoagulants (64) and patient’s visit length reduction (65). The strengths of this study include the potential to augment healthcare access in areas where specialists might not be physically available, and medication can still be prescribed utilizing the combined efforts of AI and a primary care physician, especially in developing countries. However, one missing aspect is to address the loss of privacy, as well as possibilities of patient data exploitation. The potential for Health Insurance Portability and Accountability Act (HIPAA) protection is likely feasible (1,9).

### 3.2. ML in medicine with better patient–provider interactions (32)

This approach is focused on examining the essential structural changes in the healthcare systems that are necessary to unleash the full potential of ML in medicine. It emphasizes developing the concepts of ML in medicine, which may be centralized around the idea of personalized diagnosis and treatment on the basis of all known information about the patient and collective experience. Giving rationale, authors highlighted proof-of-concept models that have been tested so far, e.g. difficulties in finding a relationship between current ML models and traditional statistical models, need for a tremendous amount of data to train ML classifiers for establishing general and complex associations and training clinicians in AI for accurate data interpretation. Furthermore, they effectively debated about consumption of physicians’ valuable time due to increased features (e.g. check boxes) in EHRs for administrative and billing purposes, which might prevent them from providing the best quality care for their patients. At the same time, they claimed that the integration of ML in EHRs may lead to potential fear of overreliance as well as decreased vigilance of errors and automation bias. Authors claimed a training ML classifier at EHRs for pattern detection to allow physicians to anticipate future events in high-risk patients, to obtain accurate and comprehensive diagnosis and provide with a quick search engine in locating the pertinent information within a patient’s chart, less clicking, voice dictation and better predictive typing.

**Table 2:** Real time examples of AI and ML algorithms (support vector machine, deep learning, logistic regression, discriminant analysis, decision tree, Random forest, linear regression, naïve Bayes, K-nearest neighbor, hidden Markov, genetic algorithm) in healthcare

**ML algorithms**	**Examples in healthcare**
Support vector machine	Symptoms classification and analysis to improve diagnostic accuracy. Identifying imaging biomarkers of neurological and psychiatric disease. Diagnosing mental illness. SVM with leave-one-out cross-validation for multiple myeloma by analyzing SNPs. SVM with 20-fold cross-validation for breast cancer by analyzing SNPs. SVM with hold-out for breast cancer by analyzing clinical, pathologic and epidemiologic data. SVM with hold-out for cervical cancer by analyzing clinical and pathologic data. SVM with 10-fold cross-validation for breast cancer by analyzing clinical and population data. SVM with cross-validation for oral cancer by analyzing clinical and genomic data. SVM with leave-one-out cross-validation for breast cancer by analyzing genomic data. SVM with cross-validation for oral cancer by analyzing clinical, molecular data.
Deep learning	Evaluating images for diabetic retinopathy. Identification of type 2 diabetes (T2D) subgroups. Measure medication adherence via camera interface. Detection and segmentation of lung and liver tumors by analyzing CT scans. Diagnosing eye diseases (diabetic retinopathy) by analyzing retinal images. Diagnosing cardiac anomalies by analyzing images of MRI of heart ventricles. Detecting malignant lung nodules by analyzing radiographs. Producing glioma survival predictions by analyzing histological imaging and genomic marker data. Histological diagnoses prediction in women with cytological abnormalities. Oncology diagnosis: **Thoracic (lung cancer)** **Abdominal and pelvic (tomography and magnetic resonance imaging)** **Colonoscopy (colonic polyps)** **Mammography (microcalcifications)** **Brain (brain tumors)** **Radiation oncology (segmenting tumors for radiation, and quantifying specific radiographic characteristics by analyzing 3D shape of a tumor from)** **Dermatology (skin cancer)** **Pathology (digital whole-slide of biopsy samples)** **Prostate (cancer tumors by analyzing ultrasound of biopsy cores)** **DNA and RNA sequencing (RNA-binding and DNA-binding proteins)**
Logistic regression	Risk assessment of complex diseases (e.g. tuberculosis, breast cancer, coronary heart disease). Predicting patient survival rate. Diagnosing coronary heart disease (CHD). Non-Hodgkin’s lymphoma diagnosis with multivariable logistic regression modeling. Identification of pulmonary thromboembolism by analyzing prognostic factors.
Discriminant analysis	Identify surgical and operative factors to classify patients for surgical procedure. Predict the clinical diagnosis of primary immunodeficiencies. Patient data satisfaction. Prediction of depression elements in cancer patients. Classification of BOLD fMRI response to naturalistic movie stimuli. Identify protein-coding regions of rice genes. Parkinson’s disease symptoms recognition. Risk assessment of for chronic illnesses. Diagnosis of hypercalcemia. Predicting patient care visits by identifying discriminatory characteristics.
Decision tree	Real-time healthcare monitoring medical decision support system, sensors anomaly detections, and data mining model for pollution prediction. Supporting clinical decisions. Strategies alternate therapies for oncology patients. Collaborative clinical decision-making in mental health care. Identify predictors of health outcomes. Find factors related to hypertension. Discover factors associated with pressure ulcers (PUs) among elderly people. Identify the potential recipients of telehealth services. Patient data stratification for interpretable decision-making for precision medicine. Content analysis for patient aids decision. Diabetic foot amputation risk analysis. Support understanding of antenatal lifestyle interventions.
Random Forest	Diagnosing mental illness. Detecting knee osteoarthritis. Monitoring medical wireless sensors. Diagnosing Alzheimer disease. Predicting metabolic pathways. Predicting outcomes of a patients encounter with behavioral health providers. Healthcare cost prediction. Mortality prediction for intensive care unit (ICU) patients. Classification of Alzheimer’s disease. Identifying social and economic factors to study social determinants of health. Predicting disease risks from imbalanced data. Identify associates of diabetic peripheral neuropathy diagnosis. Predicting the risk of emergency admission. Detecting patients ready to discharge from intensive care. Nonparametric estimation of heterogeneous treatment effects. Diagnose sleep disorders. Predicting the depression in patients suffering with Alzheimer’s disease. Predicting myopia by analyzing EHR.
Linear regression	Identification of prognostically relevant risk factors. Predict hand surgery. Monitor prescribing patterns and ensure treatment appropriateness. Mean on decision-making in health care. Reducing high costs of the health system. Analysis of skewed healthcare cost data. Understand HIV/AIDS prevalence patterns.
Naïve Bayes	Predictive modeling for different diseases (brain, asthma, prostate and breast cancer etc.). Risk prediction using censored and time-to-event data. Mucopolysaccharidosis type II detection. Predicting Alzheimer’s disease from genome-wide data. Measuring quality healthcare services. Finding audit targets in performance-based financing in health. Modeling medical diagnosis for decision support. EHR classification. Classifier and genetic score for risk prediction. Decision support system for heart disease.
K-nearest neighbor	Diagnostic performance of the model. Preserving privacy of medical diagnosis in e-Health cloud. Medical dataset classification. Classification of lymph node metastasis in gastric cancer. Pattern classification for breast cancer diagnosis. Pattern classification for health monitoring applications. Pancreatic cancer prediction combining published literature and EHR data. Disease diagnosis and detection of Parkinson. Hand-gesture recognition for elderly individuals. ECG features extraction for mobile healthcare applications.
Hidden Markov	Analyzing sequence data (predicting exons and introns, identifying ORFs, insertions, deletions, substitutions, functional motifs in proteins, aligning two sequences, and switching from exon to intron in a DNA sequence) Modeling ‘Healthy’ and ‘Unhealthy’ unobserved health states. Analyze time-series personal health checkup data. Improving length of stay prediction and reducing health care costs. Mining adverse drug reactions from online healthcare forums. Monitor, model and cluster medical inpatient journeys. Analyzing healthcare service utilization after transport-related injuries. Monitoring circadian in telemetric activity data. Predicting patients entering states with a high number of asynchronies. Analyzing subject-specific seizure, automatic segmentation of infant cry signals.
Genetic algorithm	Detecting microcalcifications in mammograms leading breast cancer. Developing non-invasive technique for cervical cancer detection. Analyzing microarray data from cancer cell lines. Investigating relationships between soil trace elements and cervical cancer mortality. Parameter estimation for determining tissue elasticity. Predicting risk of a major adverse cardiac event (MACE). Detect QRS complexes. Detecting hypoglycemia EEG signals. Predicting time to reach full cervical dilation. Selecting optimal features of cardiotocogram recordings. Identifying autism by analyzing gene expression microarray data. Predicting outcome of patients with non-small cell lung cancer (NSCLC). Diagnosing patients by classifying lung sounds into normal, wheeze, and crackle. Choosing appropriate highly active antiretroviral therapy (HAART) to control HIV. Improving the selection of gantry angles to optimize stereotactic radiotherapy. Training robot for physiotherapy of the lower limb. Estimating Cobb angle from torso asymmetry in scoliosis. Analyzing mutations in Parkinson’s disease. Predicting of tacrolimus blood levels, scheduling patient admission in ophthalmic hospital.

### 3.3. AI, ML and the evolution of healthcare (34)

Overview and implementation of AI and ML in healthcare for the extraction of big data, and aiding clinicians in providing better care delivery were recently highlighted by authors. They discussed use of support vector machine (SVM)- and deep learning-based model development for physiological data segmentation and analysis, disease progression prediction and diagnosis in radiology. Authors discussed processes, which include designing effective models to aid in the diagnosis based on the information that resemble certain diseases, image analysis and interpretation to improve the decision-making performance of clinicians. Authors also raised ethical concerns in utilizing ML, primarily in governance and management of big data and future of employment.

### 3.4. Medical AI for increasing availability of healthcare data and rapid development of big data analytic methods (39)

A study analyzed different AI applications in healthcare and discussed their potential future outcomes. Authors argued that currently AI research in healthcare is mainly focused on only a few health conditions, e.g. cancer, neural disease and cardiovascular disease. They criticized that existing healthcare systems do not provide incentives for data sharing and have no structure for the implementation of AI. Further classified applications of AI consist of ML algorithms to extract and cluster useful information from a large patient population to assist in making real-time inferences for health risk alerts and health outcome predictions, perform principal component analysis and reduce diagnostic and therapeutic errors; SVM to determine model parameters and identify imaging biomarkers; NLP for text processing and classification; and deep learning for diagnostic imaging and electronic diagnosis.

### 3.5. Data analytics and ML for disease identification in EHR (44)

Managing large volumes of data is a huge challenge for doing big data analytics. A group of researchers used an ML algorithm for structured and unstructured big data analysis to identify a wide range of medical conditions and diagnosis from the large-scale EHR database, including information about test results, historical information, management plans, and billing codes, etc. Authors initially applied their algorithm for the identification of potential predictors and keyword associations in the EHR to find presence of pseudoexfoliation syndrome (PXF) by using NLP, least absolute shrinkage and selection operator. The reported outcomes were based on positive and negative predictive values of the algorithm, which were also validated by the glaucoma specialists. Authors highlighted the potential of their algorithm with its application for data mining and predictive analytics in many other applications as well.

### 3.6. AI, big data and cancer (45)

The application of cognitive computer systems has been very successful for approaching cancer diagnosis and treatment (read, remember, recommend and remind). According to a team of researchers, cognitive computer systems can support physicians by providing rapid access to accurate information and treatment procedures, assisting in decision-making. Such systems have the potential to distribute cancer knowledge into clinical practice and remote areas worldwide, by optimizing clinical research and trials with reduced bureaucracy and cost. They presented the possibilities of AI in future cancer care and research, which includes developing international cancer networks, identifying beneficial therapies for rare and highly aggressive cancers, observing different therapeutic outcomes by different parameters, analyzing associations of cancer with other disease-specific attributes, discovering new cancer etiologies, incorporating pertinent patient and cancer characteristics into clinic-based uses, conducting economic broad-based cancer trials, uncovering genomic and molecular events sensitive to existing or new treatments and analyzing and developing new treatment pathways. Furthermore, authors predicted that large-scaled AI databases may benefit cancer programs and help cancer treatment and research.

### 3.7. Use of operational EHR in comparative effectiveness research (46)

EHR has a great role in improving the quality and cost of healthcare, advancing biomedical science and facilitating clinical research. Supporting this claim, this overview presented some examples of running and accomplished research projects, e.g. Electronic Medical Records and Genomics (eMERGE) Network, Strategic Health IT Advanced Research Projects (SHARP) Program and Health Maintenance Organization Research Network’s Virtual Data Warehouse Project. The authors also discussed multiple caveats in existing healthcare systems e.g. inaccurate data entry in EHRs; incomplete patient information in EHRs; incorrect diagnosis and medication codes (e.g. ICD, National Drug Code (NDC)), and their conversion into research descriptions and vice versa; data extraction and integration from clinical notes in to EHR; multiple data sources in EHRs affecting data provenance; mismatches of data granularity with comparative research; and differences in research protocols and clinical care.

### 3.8. Deep learning in the healthcare system (47)

Deep learning is a dominant ML approach, which has been greatly augmented in healthcare, analytic systems development and modeling tools. This review presented potential healthcare applications based on multiple driving factors, including learning digital imaging (e.g. radiology, radiotherapy, pathology, ophthalmology and dermatology) with deep learning to facilitate effective decision-making and therapy, digitization of EHR and applying ML for curating and analyzing data sets, integrative heterogeneous data analysis using deep learning, applying deep learning for hypothesis generation by identifying novel associations to establish causation and causal pathways, appropriate deployment of AI and ML-based platforms for remote monitoring and digital consultations and improving performance of deep learning with exposure to larger datasets. Authors predicted safe, effective, efficient and humanistic care in the future with the successful application of ML.

### 3.9. Deep learning to transform healthcare (48)

Deep learning has the potential to transform healthcare by outperforming clinical systems and modeling complex relationships among active hidden factors of data. This study highlights the background, workflow and challenges of deep learning with its successful implementation of digital image analysis, and some examples like analyzing fundus images of the retina to predict cardiovascular disease and identifying images for melanoma, basal and squamous cell carcinoma by matching sensitivity criteria. They suggested developing and training classifiers based on the deep neural network to make predictions on big clinical datasets by using the nonlinear features for modeling regularities. Authors claimed that in the coming years, with the continuous increase in the volume of datasets and without any enhancements to the basic learning techniques, more promising results can be achieved by ML.

### 3.10. Intelligent digital pathology with deep learning (50)

The data efficiency of deep learning can be used to augment information by improving diagnostic accuracy and efficiency by analyzing whole-slide pathology images, which cannot be easily observed by the human eye. Performance of deep learning at test sets of 129 whole-slide images was examined and compared for identifying metastases in hematoxylin and eosin–stained tissue sections of lymph nodes from women with breast cancer. Authors of the study optimized deep learning algorithms for obtaining impressive results, even better than the participating panel of 11 pathologists. They predicted a bright future of deep learning applications in digital pathology and discussed the need of new intelligent tool development for handling diagnostic sensitivity and specificity.

### 3.11. ML for predictions and finding nonlinear relationships in the EHR (51)

ML real-time applications have been used to address different clinical questions by analyzing and finding nonlinear relationships in the EHR. A research study highlighted one important aspect that could be one of the ML drawbacks and that is the utilization of most of the ML algorithms to solve clinical problems when they were originally proposed for other matrices. The authors of the study provided some examples to justify their claim and discussed that the primary purpose of operational EHR systems development is to not support any kind of ML implementation for predictions. Due to the lack of robust and structured clinical data, authors showed uncertainty in achieving high-quality results with the use of ML. They stressed the need for new tools based on the ML algorithms especially designed with prior thresholds for improvements. They also stated that the present discovery phase needs to implement ML for better understanding of heterogeneous treatment effects to implement precision medicine.

### 3.12. Analyzing and visualizing knowledge structures of health informatics to uncover the explicit and hidden patterns (55)

A quantitative review of the health informatics field uncovers the scientific growth in this field. As discussed, despite a long period of progress, there is still no proper characterization of the knowledge and no common language, which necessitates illumination of the knowledge structures, text mining methods, scientometric analysis, tracking tools, Internet of Things-based decision support systems and social network visualization to identify hidden patterns. The study conducted produced six clusters and nine research themes to support health information technology by improving patient safety by reducing medication errors, and associating decision support, knowledge representation, telehealth innovations and professional behavioral changes in medicine. For the provision of better-personalized treatment, authors suggested that the health informatics field should involve DL, new ML algorithms and advanced big data analytics.

### 3.13. Intelligent health data analytics to improve health system management, health outcomes, knowledge discovery and healthcare innovation (57)

Various healthcare systems are generating heterogeneous data of significant volumes which demands exploitation of healthcare data for resource optimization, patient satisfaction, improved care quality and health outcomes. Recommended use of AI and ML for advanced health data analytics with the arrangement of active partners in the healthcare processes requires ubiquitous services, therapeutic decision support, ethnographic health surveillance, integration of health-related data sources, personalized and predictive medicine by learning non-linear associations and drawing causal relationships among inherent data elements. A team of scientists designed a health data analytics process involving a methodical order of data processing, modeling, and analysis steps categorized as data- and knowledge-driven methods for Decision, Predictive, Descriptive, Optimization, Comparative, Prescriptive and Semantic analysis. They advocated implementation of AI and ML-based analyses with the inclusion of health data pre-processing, selecting algorithm based on expected outcome, developing analytical models and interpreting results.

### 3.14. ML in cancer prognosis and prediction (63)

ML can be applied to detect key features by predictive modeling of complex and heterogeneous datasets for progression and treatment of cancerous conditions, risks and outcomes. Many research groups have highlighted the implementation of different ML algorithms in cancer research for estimating unknown dependencies to predict new outputs of the system, including artificial neural network, Bayesian network, SVMs, graph-based semi-supervised learning (SSL) and decision tree. There are numerous ML real-world applications. The artificial neural network has been established for breast cancer by analyzing mammographic and demographic data with *k*-fold cross-validation rate and lung cancer by analyzing clinical and gene expression data; SVM has been used for analyzing single-nucleotide polymorphisms (SNPs) with leave-one-out cross-validation for multiple myeloma, with *k*-fold cross-validation for breast cancer and with *k*-fold cross-validation for breast cancer by analyzing clinical and population data; the Bayesian network can efficiently analyze clinical and pathologic data with cross-validation for colon carcinomatosis and with *k*-fold cross-validation for oral cancer by analyzing clinical and imaging tissue genomic; blood genomic data; SVM hold-out for breast cancer by analyzing clinical, pathologic and epidemiologic data and for cervical cancer by analyzing clinical and pathologic data; graph-based SSL algorithm with *k*-fold cross-validation for colon cancer; breast cancer by analyzing protein–protein interactions (PPIs) and gene expression data and *k*-fold cross-validation for breast cancer by analyzing Surveillance, Epidemiology, and End Results (SEER); SVM with cross-validation for oral cancer by analyzing clinical and genomic data; SVM with leave-one-out cross-validation for breast cancer by analyzing genomic data; Bayesian network with hold-out for breast cancer by analyzing clinical, microarray data; decision tree with cross-validation for breast cancer by analyzing SEER; SSL co-training algorithm with *k*-fold cross-validation for Breast cancer by analyzing SEER. Authors acknowledged the active contributions of ML in accurate cancer susceptibility, recurrence and survival predictions. However, some concerns exist due to lack of external validation regarding the predictive performance of models utilizing integrated clinical and genomic data. They underlined that application of ML methods in cancer requires significant validation in order to utilize them in everyday clinical practice. Authors suggested data pre-processing with focus on data modification via dimensionality reduction and feature detection.

## 4. Precision medicine approaches

The underlying assumption here is that precision medicine will provide tailored healthcare to patients and will yield lower rates of associated adverse outcomes. A classic example of precision medicine is the customization of disease treatment for a single individual which in the old paradigm was a one-size-fits-all medicine; an effective treatment is the treatment known to benefit most of the target population. However, a certain treatment may actually yield benefit to only a few individuals. The rest of the population will not benefit from the treatment and may even incur adverse effects. This exemplifies the need for AI and ML-based systems bridging multiple domains in a secure environment for heterogeneous healthcare data analysis and visualization.

### 4.1. Precision medicine with EMR analysis for prevention and treatment of diseases (33)

Different healthcare institutions do not necessarily utilize the same EMR system with the possibilities of effectively communicating with each other, which makes it difficult for physicians to track patients’ overall medical history. A team of authors brought about the importance of population perspective and need of foundation platforms for precision medicine, which can support large-scaled clinical data integration, and communication between different EMRs accessible to patients through different health centers. Authors proposed a universal EMR platform development by integrating population perspectives for the establishment of protocols to identify subgroups that fit distinct clinical phenotypes of complex disease and provides avenue for different treatment methods. Authors suggested an additional fifth ‘P’—Population-wise Perspective—to the existing 4P precision medicine concept. They justified this claim by examining a study about identification of a type 2 diabetes subgroup through a topological analysis of a population’s EMR and determined three major distinct clusters (66). Authors also suggested that it might be difficult to implement ML divisions, which include supervised learning (SVM, discriminant analysis, Naïve Bayes, nearest neighbor and neural network), unsupervised learning (linear & logistical regression, decision tree, cluster analysis and neural network) and deep learning. Authors recommended multi-cluster environment implementation for analyzing patterns within the subset of populations, which might present similar clinical phenotypes of complex diseases with the assumption that the treatment based on one cluster might not be as effective for another subgroup.

### 4.2. Solving healthcare problems with precision medicine (35)

Precision medicine has been groundbreaking in tailoring individualized and effective medical treatments based on the characteristics of each patient, and different susceptibilities to a particular disease, e.g. trastuzumab for HER2-positive breast cancer. Many authors are convinced by the importance of information technology and ML for the implementation of precision medicine, which includes data storage and analysis for determining the association between disease outcome (e.g. disease risk, prognosis or treatment), identification of patient characteristics and optimal treatment. Furthermore, authors highlight the requirement for multidisciplinary collaborations between clinicians and researchers.

### 4.3. Role of AI in patients the point-of-care, advanced analytics and foundation of precision medicine (36)

The scientific community criticizes the current healthcare structure, to be based on one-size fits all, and not transitioning from trial and error to evidence-based medicine. The authors recognize the importance of innovative technologies (e.g. genome sequencing, health sensors, advanced biotech) and essential roles of AI in precision medicine implementation. Authors proposed three-factor-based approaches, which include point of care, large-scaled clinical datasets for training classifier and analytics and finally building foundations for precision medicine. The author’s combined deep learning prediction with human pathologist’s diagnosis resulted in a success rate of 99.5% and reduced human error by 85% at an early stage. Authors also presented guidelines for implementing AI in precision medicine, which included creating ethical standards for AI applications, gradual and incremental development of AI and training medical professionals about AI.

### 4.4. Integrated precision medicine towards targeted and personalized treatment for a given patient (38)

There has been a lot of debate over the current state of clinical decision support and how it can be improved in providing precision medicine. Many scholars have discussed involving ML in healthcare settings for the diagnosis and better treatment of chronic disease in clinical, translational and public health. Furthermore, they have highlighted the significance of big data management, privacy, de-identification and data sharing. Focus on early diagnosis of chronic conditions through proper extraction of clinical insights and utilization of *in silico* datasets could allow the replacement of animal and human models when conducting clinical trials by generating virtual patients with specific characteristics that enhance the outcome of each study. According to them, 25% of drug discovery occurs by chance, which can be highly accidental. They signify using predictive, proactive intervention in healthcare through AI and clinical decision support system development for leveraging big data analysis to make better predictions on the potential outcome of patients, ultimately supporting better decision-making by the physicians.

Authors have reviewed and reported analysis of the different clinical decision support states, improvements in patient outcome and various limitations and challenges in Laboratory, Medication, Diagnosis, Radiology, Pathology, Clinical Evidence & Outcomes, and Procedures, based on the availability of different data structures and standards in healthcare systems. Challenges in the laboratory mainly involve incorporation of genetic results into EMR in a searchable way; tests conducted at external labs cannot be incorporated due to lack of standardization, not encoding lab tests with Logical Observation Identifiers Names and Codes (LOINC), missing genetic information in EHR. Challenges in Medication include over time and ineffective drug combination. Challenges in Diagnosis surround handling of International Classification of Diseases (ICD) codes, as not all codes are not billable and some diagnoses are not even encoded. Challenges in Radiology are of metadata mostly not formatted according to Digital Imaging and Communications in Medicine (DICOM), and reports are unstructured. Challenges in Pathology hold generation of unstructured reports, and frequent utilization of unstandardized nomenclature. Challenges in Clinical Evidence & Outcomes consist of insufficient data types, and unavailability of universally adopted data models. Challenges in Procedures mainly cover the process for approving new procedural codes. Authors have discussed the evolution of EHR to effectively delivering personalized treatment. With discussion of two case studies, they emphasized the implementation of precision medicine for the personalized delivery of care based on patient-specific patterns of disease progression and determination of precise therapies.

### 4.5. ML knowledgebase with ontology for pattern recognition in personalized medicine (40)

Personalized medicine is a broad and rapidly advancing field in healthcare, primarily based on disease-related clinical, genomic, metabolomics and environmental information. However, failure to correctly identify disease is one of the major reasons for misleading diagnosis, treatment and prognosis for the patient. A research group presented essential components (pattern recognition, knowledge base, ontology and patient profile) for accurate disease examination needed for successfully integrating personalized medicine into everyday clinical practice. Utilizing ML approaches for pattern recognition and development of statistical models (sample size and effect size), creating a knowledgebase of all existing phenotype categories and disease, organization of clinical datasets of population size and open software platform development for statistical analysis of high-dimensional healthcare and multi-omics data are crucial for practical realization of precision medicine.

### 4.6. ML classifies cancer by visual assessment of tumor cells (54)

One of the key technological advancements in the diagnosis of brain tumors was microscope-based analysis. Overcoming the visual limitation of such techniques for leading different classifications of a given sample by different individuals, a group of authors discussed ML as a precise solution for accurate diagnosis by analyzing molecular data. A previous study trained an ML classifier at a maximum number of images of tumors that were classified by physicians, as it is not possible to get precise conclusions, especially when tumor is histologically indistinguishable. Authors discussed the application of supervised ML for analyzing central nervous system (CNS) tumor-type genome-wide methylation data to identify methylation patterns. They mentioned application of unsupervised ML to search patterns in the data sets to develop classification categories. Authors suggested application of ML for molecular analysis and visual inspection, as the disease can be the manifestation of both molecular and cellular changes.

### 4.7. ML and genomics in precision medicine (62)

Authors studied major concerns regarding data security like breaches in patient privacy and also presented the latest developments in the field of computational data protection. They discussed advancements in the era of big data for computer-aided diagnosis, and a recent concept of precision medicine for providing care for clinical, environmental and genetic characteristics. Authors emphasized developing new ML algorithms for computational analysis of genomic data to systematize the process of finding genetic similarities among patients with similar prognosis, or response to a treatment. They also stressed the need of implementing intelligent procedures to ensure the privacy of data, as they believe that data anonymization is not enough to guarantee unidentifiability due to auxiliary information. They discussed some existing solutions, which includes de-identification by data suppression, k-anonymization, learning from noisy data, homomorphic encryption, multi-party computation, cryptographic hardware and protecting genomic databases. Authors acknowledged the current contributions of ML but with the expectations for substantial improvements to address clinical data security problems and development of new significant ML models to address the challenges of gene variations and similarities among patients. They suggested combining the latest computational data protection principles with legal and ethical perspectives to construct a secure framework for data sharing.

## 5. Healthcare resource management and ethical challenges

Resource management is very important in any field of life, especially in healthcare. Aligning people and technology with organizational goals can positively impact with efficient implementation of planned workflow in achieving on time high-quality results. However, inefficient resource management may lead to over-exaggeration of organizational resources, which includes, time, cost, manpower and computational, bench and infrastructure resources. Furthermore, it is important to address the ethical and data privacy challenges, when implementing traditional state-of-the-art and intelligent healthcare data analytics.

### 5.1. AI towards health in resource-poor settings (37)

Importance and utilization of AI to improve health outcomes in low-income settings and regions has always been a point of discussion. One research focus has been on clearly identifying problems and issues related to the integration of global healthcare, application and deployment of AI in real-time environments, high-quality healthcare data collection in under-developed areas, automatic data extraction from hand-written notes in local languages, availability of high-performance computing and cloud-based environments for data management and analysis, and construction of knowledge bases and expert systems (67). Furthermore, concerns about data security, consent, and ownership, and ethical, patient safety and privacy-related issues that accompanies the utilization of AI are also present. Many solutions have been presented to address the problems of implementation of NLP in EHR for surveillance and predictions, AI implementation at pre-existing systems, weather and land pattern identification and tracking disease transmission through ML algorithms, implementing advanced expert systems, utilizing NLP to translate hand-written notes according to WHO standardized medical terminologies and developing local dictionaries, establishment of environments capable of working offline and synchronized with the remote databases, adopting cloud computing for the implementation of public health without established IT structure in Low and Middle Income Countries (LMICs), and using ‘blockchain’ for cryptocurrency and addressing issues related to the privacy and transparency. The scientific fraternity has predicted tremendous cost-saving and improved care delivery in coming years with the implementation of such along with related tasks.

### 5.2. Data science, AI and ML for laboratory medicine (41)

Data science has demonstrated success in laboratory medicine and reinforcing its value in transforming the healthcare system. This is a review on the perspective applications of data science current problems in healthcare, which includes the need of significant computational power to contribute to solving data optimization problems, overfitting in experimental designs, lack of data standardization, large-scaled datasets for training ML classifiers and other concerns in clinical laboratories, e.g. protected health information challenges, financial limitations and ethical concerns. Authors discussed the concept that data science and AI mimics human processes and improves the process of decision-making. They reasoned the use of predictive modeling for better collaboration between hospitals without sharing data and complying privacy regulation through ML (supervised and unsupervised learning) for healthcare data (clinical data, imaging, laboratory tests) mining, analysis and optimization, identification of large patient clusters with certain disease characteristics, reducing medical errors and cost and improving efficiency of staff and resources. Data science has its own applicability and ethical challenges especially related to AI integration. AI encompassing data science when adopted can even improve human thought processes in efficient decision-making with different ML algorithms, data mining, knowledge discovery, improving complex analytical tasks and calculating clinical pathways. Authors of a study acknowledged success stories of ML in healthcare (radiology, pathology, dermatology and genomics), weather forecasting, structural recognition, NLP, games, analyses of financial transactions and improvements in industrial processes by finding patterns, discovering inefficiencies, predicting outcomes and taking factual decisions. They provided examples of ML application in healthcare for the detection of cervical cancer (neural networks), prediction of histological diagnoses (artificial neural network-based decision-support scoring systems) and radical hysterectomies with gene expression analysis (neuronal network), DNA (epigenetic modifications) and RNA (messenger, long non-coding or double-stranded) data analysis. ML is contributing to better personalized treatment by monitoring patient activities (analyzing data received by sensors for continuous measurement, e.g. glucose); analyzing molecular biomarkers; predicting drug efficacies, treatment responses and disease pathways; and identifying molecular factors and genetic variants. The authors presented an ML framework, which includes defining tasks, matrices, models and datasets. They showed concerns regarding implementation of human rights by the Universal Declaration of Human Rights at the 1948 United Nations General Assembly for data privacy, protection, de-identification and encryption for data handling, collection and sharing. Authors emphasized the consideration of key ethical concerns, which include consenting patient, AI human warranty and regulation of healthcare data according to principles of bioethical law.

### 5.3. AI to solve the human resource crisis in healthcare (42)

The healthcare workforce crisis is widening across the globe. With the increase in the number of chronic and complex diseases, the demand towards efficient healthcare system is consistently growing. However, the lack of access to care, differing quality and doctor shortages are increasing worldwide. Some authors have presented AI as the solution in healthcare and discussed its progress in the direction of predictive and proactive interventions in clinical decision support systems. They reviewed AI approaches, dividing them into three categories (43), artificial narrow intelligence (performing a single task), artificial general intelligence (agent-based system, known as human-level AI) and artificial superintelligence (agent-based system, but smarter than better human). Authors debated the potential role of AI in filling the human resource gaps by integrating it with physicians to improve diagnostics and help in better decision-making. At the same time, they also highlighted the ethical implication of using AI technology as integrating part of the healthcare system.

### 5.4. High-performance medicine with AI for improving workflow and reducing medical errors (49)

There are multiple challenges of implementing AI in medicine. A research group underlined the obstacles and caveats, especially when applied in radiology, pathology, dermatology, ophthalmology, cardiology, gastroenterology and mental health. They predicted adaptation of AI by almost every type of clinician, which mainly includes deep neural networks for pattern recognition and analysis of medical images (e.g. medical scans, pathology slides, skin lesions, retinal images, electrocardiograms, endoscopy, faces and vital signs); applying deep learning to EHR for estimating the risk of a patient’s hospital readmission; supporting doctors in decision-making for resuscitation; determining patients at risk of developing sepsis and other diseases; and predicting biological age, and critical diseases leading to death. Authors highlighted current challenges to the field of life sciences that can be solved by ML algorithms, like identification and isolation of rare cells, multi-omics data analysis, classification of somatic and germline mutations and gene–gene interactions and prediction of protein structure and PPIs, the microbiome and single cells. They also discussed the use of NLP in drug discovery by analyzing biomedical literature; mining molecular structures; predicting off-target effects, toxicity and right dose for experimental drugs; developing cellular assays; and using AI cryptography for determining unidentified drug interactions. Together with these advantages, authors also discussed formidable obstacles and drawbacks due to the field of AI, which includes data privacy and security, data hacking and breaches, uncertainty in use of black boxes of algorithms to resolve output, state of AI hype in validation and readiness for implementing in patient care. Authors anticipated the bright future of AI applications by predicting useful clinical outcomes in health systems, algorithmic interpretation of images and data, reducing errors, inefficiencies and cost.

### 5.5. Big data and ML algorithms for better healthcare delivery (56)

ML is considered a branch of AI encompassing algorithmic methods to solve scientific, healthcare and various other problems without traditional computer programming; this involves interplay between large population-specific datasets and model building. Suggested AI tool development should be based on incremental learning (ability to continuously improve with the inclusion of new data) to refine predictive accuracies. One study proposed analytics exercise starting with the identification of clinical problems, annotation of extracted healthcare data, application of appropriate ML algorithms and its effect on decision-making, addressing legal and ethical implications, assessment of ML effect in trial, designing freeze and submission of dossier for medical devices, training clinicians on use of ML tools and monitoring for adverse outcomes. Authors of the study presented ML algorithms suitable for different clinical applications and reported real-time clinical developments in oncology with the implementation of different AI and ML applications and algorithms. Authors discussed the importance of human–computer interaction in AI and ML platform development to effectively support decision-making processes in healthcare. They presented different real-time examples, which include HealthSuite, GE Healthcare, Lumada, DISCOVERY and CURATE.AI. They recommended model linking EHR in future ML platform development for addressing healthcare data analytics concerns.

### 5.6. Causal inference with ML (60)

Another study examined the implications of progress of AI in observational research design and healthcare databases, and implementation of ML for data classification and prediction in Real-World Evidence (RWE) to support clinical and regulatory decision-making. They discussed American Recovery and Reinvestment Act (ARRA) explicitly prohibiting cost-effectiveness and focusing on broad interventions for better diagnoses, treatments, disease management programs and healthcare organization models by mainly analyzing EHR and establishing patient-centered Outcomes Research. Authors discussed a wide range of increased size and nature of biomedical operational and research data, which requires efficient utilization of ML approaches to restructure, dimensionality reduction, clustering, modeling, linking, classification, analysis and predictions. Meeting RWE objectives, authors acknowledged ML as one of the powerful tools today for bringing significant improvement to care service. They exemplified ML with the implementation of SVMs to predict hospitalization; regression-based methods for reducing the risk of overfitting; *k*-fold cross-validation for splitting one’s sample into two models; and deep learning models for feature extraction, development of graphical processing units, predicting in-hospital mortality, unplanned readmissions, prolonged stays, and discharge diagnoses. However, they showed lack of confidence in ML for shielding against the normal challenges in observational data analysis, as screening some concerns require evidence.

### 5.7. Promise and potential of big data analytics in healthcare (61)

The effective use of big data by digitizing, combining large hospital networks and implementing efficient analytical approaches has been widely successful. According to a team of scientists, there are tremendous benefits of such approach in detecting diseases at earlier stages; predicting risk for medical complications; managing individual and population health, avoiding frauds; and addressing numerous healthcare questions. Furthermore, it can reduce waste and inefficiency by determining clinically relevant and cost-effective ways to diagnose and treat patients, applying ML algorithms to predict models and analyzing EHR and disease patterns to discover adverse treatment effects. Authors also discussed the current challenges, covering unavailability of user-friendly and transparent real-time big data platforms; lags between data acquisition, collection, cleansing, processing and standardization; missing ability to manipulate data at different levels for granularity, privacy and security enablement and quality assurance; difficulties in the management of large, diverse and complex data with traditional approaches; changing healthcare reimbursement models; lack of professional tools, infrastructure and techniques to leverage big data effectively; and absence of dynamic analytics algorithms for efficient data modeling. Authors presented a conceptual architecture of big data analytics, from developing multi-source data input, transformation, structure, management and analysis using traditional SQL, OLAP, to data mining.

### 5.8. Unintended consequences of ML (52)

Authors reviewed some of the factors driving wide adoption of deep learning and other forms of ML in the health ecosystem. They stated that ML has the potential of promoting changes in specialty that requires accurate prognosis models (e.g. oncology) and pattern recognition (e.g. radiology, pathology). However, authors anticipated some unintended consequences due to the application of ML-based decision support systems in healthcare, which includes overreliance on automation, more potential for decision errors by physicians, misleading diagnosis, misinterpretation of data, intrinsic uncertainty in medicine, inevitable intrinsic uncertainties and rationale and inscrutable outcome of ML algorithms. Authors presented unintended consequences in clinical and operational research support in reducing the odds and better implementing ML in medicine. Authors appealed for developing more precise analytics platforms for pathology images, next-generation radiology tools, converting EHR into reliable risk predictors and monitoring patients’ health through wearables and personal devices.

### 5.9. Finding the missing link for big biomedical data (53)

The problem of integration and analysis of large-scale biomedical data located in heterogeneous data sources can be solved by intelligently conforming biomedical data to support physicians and researchers in conducting new studies and drawing new hypothesis, leading to novel interventions. This study lists challenges in biomedical data linking, which includes identification of potential sources, determination of linking values, lack of a national unique patient identifier and data privacy and security concerns. The authors also presented some potential solutions to these challenges and support personalized treatment, urging the identification of potential sources of health information, considering non-traditional data (e.g. social media, purchase history, census records, etc.) to assemble a universal view of a patient and probabilistic linkage to the unique patient identifier problem. They recommended future (AI and ML) tools development to analyze biomedical data for better clinical decision-making.

### 5.10. Ethical challenges of implementing ML in healthcare (58)

The current trend towards AI and ML algorithm development and application comes with its set of issues and challenges. Despite the helpful contributions of AI and ML in healthcare, they have some concerns, especially related to the ethical challenges, which include unexpected risks due to the utilization of algorithms in medicine originally proposed for some other development; likelihood of using ML as the source of the communal medical mind; mirroring human prejudices in decision-making by learning from different unforeseen biases; in extreme premature conditions relying on ML results can lead to fatal conclusions; mostly applied ML algorithms are designed to perform in unethical conditions; ethical strain of gaining industrial profits with clinical decision-support systems without informing its users; increase in ethically problematic outcomes due to constructed ML black boxes; and reimagining of confidentiality due to ML. A review justifies these limitations in AI and ML with some examples, e.g. Uber’s software tool Greyball and Volkswagen’s algorithm. Authors are hopeful that these issues will be addressed in future ML-based healthcare systems, with the involvement or professionals and researchers from policy enactment, programming, task-forces (59), etc.

## 6. Theoretical background of AI, ML and examples in healthcare

AI aimed to improve the intellectual capabilities and performances of machines to solve complex and big data-oriented problems by classifying interaction patterns among variables, learning from experiences, strategizing and predicting better orientations. AI has been in business for almost over 50 years, and its applications are heavily demanded in numerous fields of life, science, technology and medicine (68,69). AI developments are based on supervised, unsupervised and reinforced learning principles, which include computational command line, desktop, web-based, robotics and smartphone applications with different analytics capabilities, e.g. machine translation, speech recognition, NLP, data mining, risk modeling, image recognition, machine vision, knowledge bases, expert systems and agent-based systems. AI is categorized in four main types for decision-making, which are reactive machines (based on the current situation without learning from experiences), limited memory (based on short memory, and learning from experiences), theory of mind (based on humanlike capabilities and abilities to attribute mental states) and self-awareness (based on human-level consciousness).

In the last few years, AI has become more popular and seriously considered for analyzing diverse clinical data (EHR, images, etc.) for accurate diagnosis and effective treatment in different practices (70), e.g. radiology (71) (early diagnosis, enhance visualization of pathologies and predicting emergency situations (64,73,71)), oncology (diagnosis of breast (75), skin (76), lung cancer (77)), cardiology (interpreting electrocardiogram readings, echocardiography with 3D cardiac imaging, cardiac CT angiography for calcification of the coronary vessels, cardiac MRI for measuring perfusion and blood flow and longitudinal evaluation to find predictors of heart failure (78,79,80)), gastroenterology (analyzing endoscopic images for screening regimens to abnormal findings (81)), ophthalmology (detection of diabetic retinopathy in retinal fundus photographs (64)), pediatric (augmenting diagnostic evaluations (82)) and surgery (robotic-assisted surgery (83,84)), but not limited to these (85). One of the most recent trends is the utilization of AI in Precision Medicine (86,87,88) with the application of ML algorithms for analyzing heterogeneous patient data, e.g. clinical, genomics, metabolomics, imaging, claims, labs, nutrients and life-style.

**Figure 2:**
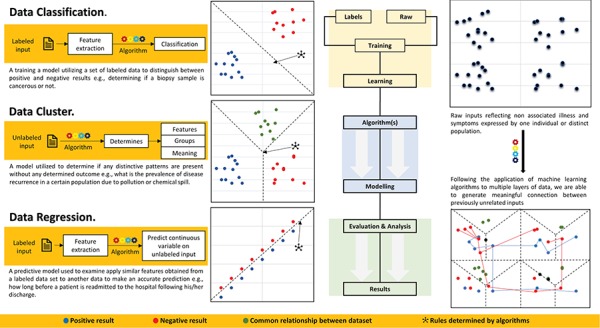
Data classification, clustering and regression for healthcare data analytics. ML application process includes creating and labeling of raw data, training classifier for data modeling using appropriate algorithm and analyzing and reporting results.

ML is a branch of AI that utilizes and proposes different algorithms for learning from numerous data variables and revealing multifaceted relationships among data features to predict accuracies in different contexts and support decision-making processes. The overall ML process starts with the manifestation of either or both data and labels, training classifier to learn and model using an algorithm and then performing data evaluation and analysis to estimate final results. ML is mainly categorized in three learning approaches: classification, cluster, and regression (Figure 2). Classification and regression are based on supervised learning, while clustering involves unsupervised learning. Classification predicts discrete, categorical response values by using labels and parameters, e.g. determining if a biopsy sample is cancerous or not (positive/negative). Cluster is to partition data into sub-groups, e.g. what is the prevalence of disease recurrence (positive/negative) in a certain population due to pollution or chemical spill (common relationship between dataset). Regression predicts continuous-response numeric values to identify distribution trends, e.g. how long before a patient is readmitted to the hospital following his/her discharge (positive/negative).

**Figure 3:**
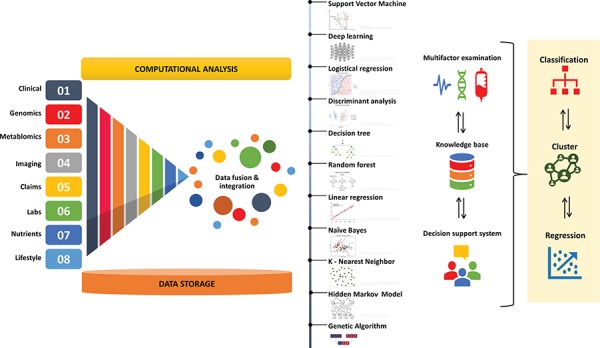
Applying machine learning algorithms for clinical, genomics, metabolomics, imaging, claims, labs, nutrients and life style data fusion, integration and analysis. Machine learning algorithms include, support vector machine, deep learning, logistic regression, discriminant analysis, decision tree, Random forest, linear regression, naïve Bayes, K-nearest neighbor, hidden Markov model and genetic algorithm.

ML is becoming the transformational force in healthcare (89) for guiding individual and population health with several computational benefits, which includes real-time patient monitoring; disease patterns analysis (90); disease diagnosis and prescription of medicines; patient-centric care provision with enhanced treatment (91); clinical errors reduction (92); prognostic scoring (93); therapeutic decision-making (94); identification of sepsis and high risk for medical emergencies (95); identification of phenotypes; screening claims data (96); extraction of clinical codes from death certificates and autopsy reports; identification of heart failure, cancer and other chronic disease causing symptoms; risk predictions, interventions, paneling and resourcing (97,98) and clinical decision-making (99). Most commonly used ML algorithms in medicine includes SVM, deep learning, logistic regression, DA, decision tree, random forest, linear regression, Naïve Bayes, K-nearest neighbor (KNN) and hidden Markov model (HMM) (Figure 3).

SVM (100) is one of the most widely used ML algorithms in bioinformatics and healthcare (101). It is considered a great supervised learning method (102,103) for accurately working in general practice. SVM was proposed in 1963 (100) to model diverse and high-dimensional data (101) with kernel methods to generate nonlinear decision boundaries and train classifier (104,105,106). It assists in the field of medicine with vast variety of contributions, e.g. symptoms classification and analysis to improve diagnostic accuracy, identifying imaging biomarkers of neurological and psychiatric disease, validation for multiple myeloma and breast cancer by analyzing SNPs, hold-out for breast and cervical cancer by analyzing clinical, pathologic and epidemiologic data, validation for oral cancer by analyzing clinical, molecular and genomic data, and diagnosing mental illness (106,107,108). Its ML benefits include modeling nonlinear class boundaries, unlikely overfitting, reduced computational complexity to quadratic optimization problems and controllable complexity of decision rule and frequency of error (109). However, the complex structure of this algorithm is its limitations leading to slow data processing speed, and difficulties in determining optimal parameters especially when training dataset is not linearly classified (108).

Deep learning is a dominant approach based on artificial neural network. It was inspired by the ability of the brain to learn complicated patterns, and one of the most popular ML algorithms in healthcare today, aiming to advance clinical medicine and care delivery (47). The artificial neural network base gives its several nested layers of neurons (110) to learn complex relationships between features and labels from heterogeneous clinical data (32). Deep learning has four categories: deep belief network, deep neural network, convolutional neural network and recurrent neural network (39) for implementing pattern recognition and predictive modeling at high-dimensional big data sets. It is fundamentally a uniquely different paradigm in ML (111,112) and its capabilities include multitasking, automatic construction of complex features, digitization of EHR and image-based data, integrating heterogeneous data sets assembled from diverse sources, combining with wearables for remote monitoring (47). Its ML benefits include application in classification or regression with ability to represent Boolean functions (AND, OR, NOT), handling noisy inputs and classifying instances for more than one output. However, its limitations include difficulties in understanding structures of algorithms, possibility of too many overfitting attributes and its optimal network structure which can only be created by experimentation (108). Deep learning is one of the most widely used algorithms in medicine for analyzing different types of images from various healthcare disciplines but especially oncology, e.g. thoracic (lung cancer); abdominal and pelvic (computed tomography (CT) and magnetic resonance imaging (MRI)); colonoscopy (colonic polyps); mammography (microcalcifications); brain (brain tumors); radiation oncology (segmenting tumors for radiation, and quantifying specific radiographic characteristics by analyzing 3D shape of a tumor (113,114)); dermatology (skin cancer (115,76)); pathology (digital whole-slide of biopsy samples) (115); prostate (cancer tumors by analyzing ultrasound of biopsy cores); malignant lung nodules by analyzing radiographs; glioma by analyzing histological imaging and genomic marker data; and DNA and RNA sequencing (RNA-binding and DNA-binding proteins). Furthermore, deep learning has been applied for the diagnosis of several other diseases, e.g. nodular BCC, dermal nevus and seborrheic keratosis in dermatopathology (116); diabetic retinopathy (64); type 2 diabetes subgroups (66); diabetic retinopathy by analyzing retinal images; histological prediction in women with cytological abnormalities; measure medication adherence via camera interface (65); and cardiac anomalies and congestive heart failure by analyzing images of MRI of heart ventricles (117).

Logistic regression is a statistical method to assess the relationships between various predictor categorical and continuous variables and dichotomous binary outcome (118). It has been applied in the fields of medicine (clinical practice, surgery), epidemiology and biochemistry to perform predictive and explanatory modeling by obtaining odds ratios and risk factors explaining variations in specific outcomes (119,120,121,122). Its examples of real-time implementation in the field of medicine include risk assessment of complex diseases (123) (e.g. tuberculosis (124), breast cancer (125)), predicting patient survival rate and diagnosing coronary heart disease (CHD) (123), non-Hodgkin’s lymphoma diagnosis with multivariable logistic regression modeling (126), and identification of pulmonary thromboembolism (PTE) by analyzing prognostic factors (127). Its ML benefits include concurrent multiple explanatory variables analysis with reduced effect of confounding factors and modeling categorical dependent variables (128,129). However, its limitation lies in handling continuous explanatory variables with more than two levels as it is based on variables with a constant range of values (122), difficulties in understanding odds and probabilities (119), right predictor variable selection (118,122), reference group setup and managing relationships between input and output variables.

Discriminant analysis is a widely applied technique in medical studies for pattern recognition (130). Discriminant analysis is used to predict and classify group members by building one or multiple functions (131,132,133) based on normal distribution and equal variance–covariance of independent variables (134,135). Its real-time applications include identifying surgical and operative factors to accurately classify patients for surgical procedure (136), predict the clinical diagnosis of primary immunodeficiencies (137), patients’ symptom-relief satisfaction data (138), prediction of depression elements in cancer patients (135), classification of BOLD fMRI response to naturalistic movie stimuli (139), identify protein coding regions of rice genes (140), Parkinson’s disease symptoms recognition (141), risk assessment for chronic illnesses (142), diagnosis of hypercalcemia (143) and predicting patient care visits by identifying discriminatory characteristics (144). Its ML benefits include robustness, reduced dimensionality, easy implementation as it requires fewer parameters to be estimated (137). However, its limitations include over-fitting to dataset (131), limit performance at novel datasets (145) and lack of cross-validation (137).

The decision tree utilizes a tree structure modeling approach with conditional control statements for establishing an efficient decision-making process (146). The decision tree is based on the concept of classification rules, following paths from root to leaf. Its internal nodes represent ‘test’ on an attribute, branch represents the outcome of the test and leaf represents decision taken after computing all attributes. Its ML benefits include ease of understanding, order of training algorithm instances with no effect, and no overfitting problem while pruning, allowing predictive model implementation with high precision, permanence and ease of clarification, well-mapped non-linear relationships and suitability for both classification or regression problems. However, its limitations include mutually exclusive classes, dependency on order of attribute selection, being error-prone when training on excessively complex decision tree, time-consumption and branching of missing values for an attribute (108). It has been well applied in the field of medicine for real-time healthcare monitoring, medical decision support system, anomaly detecting and sensor and a data mining model for pollution prediction. A few real-time examples include supporting clinical decisions (147), strategies for alternating therapies in oncology patients (148), collaborative clinical decision-making in mental health care (149), identifying predictors of health outcomes (150), finding factors related to hypertension (151), discovering factors associated with pressure ulcers (PUs) among elderly people (152), identifying the potential recipients of telehealth services (153), patient data stratification for interpretable decision-making for precision medicine (154), content analysis for patient aids decision (155), diabetic foot amputation risk analysis (156) and support understanding of antenatal lifestyle interventions (157).

Random forest is also known as the random decision forest, a combination of algorithms to build predictive models for classification and regression problems (158). Its classifier generates a set of decision trees based on training of randomly selected subsets to aggregate the elects from different decision trees to get final object. Its ML benefits include overcoming the problem of overfitting, less variance, not requiring input data preparation, flexibility and high accuracy even with missing large portions of the data. However, its limitations include its complexity, difficulty in implementing, requirements for additional computational resources, less intuitiveness and more time consumption than most other algorithms. Nevertheless, it has been applied in the field medicine for data mining, real-time patient monitoring, disease classification, implementation in wearables and personal devices and modeling big data based on engine recommendations. Some of its reported contributions include diagnosing mental illness (106), detecting knee osteoarthritis (159), monitoring medical wireless sensors (160), diagnosing Alzheimer’s disease (161), predicting metabolic pathways (162), predicting outcomes of a patient’s encounter with behavioral health providers (163), healthcare cost prediction (164), mortality prediction for intensive care unit (ICU) patients (165), classification of Alzheimer’s disease (166), identifying social and economic factors to study social determinants of health (167), predicting disease risks from imbalanced data (168), identifying associates of diabetic peripheral neuropathy diagnosis (169), predicting the risk of emergency admission (170), detecting patients ready to discharge from intensive care (171), nonparametric estimation of heterogeneous treatment effects (172), diagnosing sleep disorders (173) and predicting depression in patients suffering from Alzheimer’s disease (174).

Linear regression is an ML approach to model relationships between dependent and independent variables using linear predictor functions to identify errors of prediction in a scatter plot and characterize relationships among multiple factors (175). Linear regression has been applied in the field of medicine for many computational analyses and predictions, from identification of prognostically relevant risk factors (175), predicting hand surgery (176), monitoring treatment prescribing patterns and ensuring its appropriateness (177), averaging decision-making in healthcare (178), reducing high costs of the health system (179), analyzing skewed healthcare cost data (180) and understanding human immunodeficiency virus (HIV) prevalence patterns (181).

Naïve Bayes is a supervised ML technique based on Bayes’ theorem for data mining, classification, and predictive modeling (182) to find a maximum probability value from a conditional probability chain (183). Naïve Bayes is well applied in the field of health performing predictive modelling for different diseases (brain, asthma, prostate, and breast cancer etc.) (184), predicting risk using censored and time-to-event data (185), detecting Mucopolysaccharidosis type II (186), predicting Alzheimer’s disease from genome-wide data (187), measuring quality healthcare services (188), finding audit targets in performance-based financing in health (189), modeling medical diagnosis for decision support (190), classifying EHR (191), classifying and genetic scoring for risk prediction (192), and designing a decision support system for heart disease (193). Its ML benefits include modelling based on statistical foundation, ease to understand and train algorithm, and usefulness across multiple domains. However, its limitations include difficulties in handling redundant attributes, distribution of statistically independent attributes, and management of class frequencies affecting accuracy (108).

KNN is also a supervised learning-based algorithm used for classification and regression in pattern recognition, data mining and intrusion detection by classifying points to given categories from a training dataset (194). Its ML benefits include implementation of instances for fast non-linear data classifications, robustness in addressing irrelevant or novel attributes, well-handled instances with noise and missing attribute values and applicability for both regression and classification. However, its limitations include languidity in updating description concept, expecting similar classifications and relevancy from instances with similar attributes and increase in computational complexity with the number of attributes (108). KNN has been used in many scientific fields but with limited applications in the field of medicine (195). Some of real-time examples include modeling diagnostic performance (195), preserving privacy of medical diagnosis in e-Health cloud (196), medical dataset classification (197), classification of lymph node metastasis in gastric cancer (198), pattern classification for breast cancer diagnosis (199), pattern classification for health monitoring applications (200) and pancreatic cancer prediction combining published literature and EHR data (201).

HMM was originally proposed to solve speech problems by making complex and instinctive probabilistic models for finding and processing hidden states and paths (202,203,204). Since the late 1980s, it has been effectively applied in the field of life sciences, especially in biology for analyzing sequence data (e.g. predicting exons and introns, identifying ORFs, insertions, deletions, substitutions, functional motifs in proteins, aligning two sequences and switching from exon to intron in a DNA sequence (205)) by capturing hidden information from observable sequential symbols. Later, it was well adapted in the field of medicine and its real-time contributions include modeling ‘Healthy’ and ‘Unhealthy’ unobserved health states (206); analyzing time-series data on personal health check-up (206); improving length of hospital stay prediction and reducing health care costs (207); mining adverse drug reactions from online healthcare forums (208); monitoring, modeling and clustering medical inpatient journeys (209); analyzing healthcare service utilization after transport-related injuries (210); monitoring circadian in telemetric activity data (211); predicting patients entering states with a high number of asynchronies (212); and analyzing subject-specific seizure and automatic segmentation of infant cry signals (213). However, its modeling limitations include computing probability of sequence observation, choosing an accurate corresponding state, adjusting parameters (214) and dealing with correlations between residues due to the underlying decency assumption problem (215).

The genetic algorithm was inspired by Charles Darwin’s theory of natural evolution to solve constrained and unconstrained data optimization and standardization problems by repeatedly modifying a population of individual solutions (215). Primarily, it is based on heuristic search with three active rules: selection of data elements (parents), crossover rules for two parents from children and mutation for random change (216). Its ML benefits include easier implementation than other algorithms; application for feature classification, selection and optimization; and relative success. However, its computational limitations include development of non-trivial scoring function, complications in training classifiers of given data, and its being not the best method to find optima. It has been vigorously involved in the fields of life and medical sciences for benefitting analytics, development in radiology, oncology, cardiology, endocrinology, pediatrics, surgery, pulmonology, infectious diseases, radiotherapy, rehabilitation medicine, orthopedics, neurology, pharmacotherapy, health care management, obstetrics and gynecology (217). It has been successful in detecting microcalcifications in mammograms of breast cancer (218,219), developing a non-invasive technique for cervical cancer detection (220), analyzing microarray data from cancer cell lines (221), investigating relationships between soil trace elements and cervical cancer mortality (222), parameter estimation for determining tissue elasticity (223), predicting risk of a major adverse cardiac event (MACE) (224), detecting QRS complexes (225), detecting hypoglycemia EEG signals (226), predicting time to reach full cervical dilation (227), selecting optimal features for cardiotocogram recordings (228), identifying autism by analyzing gene expression microarray data (229), predicting outcomes of patients with non-small cell lung cancer (NSCLC) (230), diagnosing patients by classifying lung sounds into normal, wheeze and crackle (231), choosing appropriate highly active antiretroviral therapy (HAART) to control HIV (232), improving the selection of gantry angles to optimize stereotactic radiotherapy (233), training robots for physiotherapy of the lower limb (234), estimating Cobb angle from torso asymmetry in scoliosis (235), analyzing mutations in Parkinson’s disease (236), predicting tacrolimus blood levels and scheduling patient admission in an ophthalmic hospital (237).

## 7. Discussion

AI and its application in healthcare could be another great leap in medicine and a transformational force for guiding personalized and population medicine with several computational benefits. The extent of its popularity in healthcare can be easily determined by the number of AI related published work in medicine. At the time of the study (09 April 2019), a total of 16 166 AI and ML papers were available through PubMed. The growth of scientific literature in AI increased in the last 10 years with 14 469 papers (2009–2019), and more than 70% work was published in the last five years. While the growing importance and relevance of AI in healthcare is indisputable, to improve public sector clinical practice, there is a critical need for development of intelligent frameworks to connect operational and analytical healthcare systems in a way that experts from multiple domains can perform measurement and predictive analysis. AI has the potential to play a vital role at various levels of clinical operations, research and analytics to achieve significant improvements in providing better individualized and population healthcare at lower costs, and together with better work-life style for clinicians and staff. The major barriers in successfully implementing AI in healthcare include gaps in AI building blocks and infrastructures; low quality data availability; business model sustainability; regulations and policies for data collection, usage and sharing; disruption in the physician–patient relationships; integration to operational health systems; reduced evidence and reproducibility; selection of most appropriate ML algorithm; lack of understanding of AI and ML processes to predict; hazard of dehumanization of healthcare data and job insecurity threat; conflicts of interest and impartial access; accountability exploitation of AI; ‘sanity’ check to minimize any bias; handling of misleading and erroneous results; and data privacy, ethics, consent and ownership (108,238,239,240).

Determining which AI approach to use for which task is a challenge in itself. Classifying tasks based on available predictor variables is a key step to correctly addressing the problem. Traditional AI models can be used for simple prediction tasks while complex tasks require more complex models. The next step is to consider how the model will be used in practice. Setting rules to identify if a task needs to pre-process data before execution or if a complex task can be broken down into simple tasks is amenable to a traditional model. Creating and updating these rules is very time-consuming but is very useful. Availability of training data is another key factor in intelligent automation. Simple models can work with little data and few variables, but complex models require huge amount of data with multiple examples and scenarios as it is expected to remove noisy data and learn to identify complex statistical patterns (32). Numerous approaches have been proposed recently to successfully target current challenges of implementing AI and ML. We studied and reviewed contributions and variability analysis of various approaches in healthcare. We defined 15 different features to assess the potential of discussed approaches (Table 3). These features are (i) intelligent interface development; (ii) next-gen radiology and imaging tools development; (iii) global expansion of medical resources; (iv) automated ETL, linkage and data mining in HER; (v) risk prediction and containment of antibiotics resistance; (vi) pathology image analysis; (vii) AI in machines and medical devices; (viii) smart solutions and methods for cancer treatment; (vix) EMR analysis for accurate risk predictors; (x) wearable devices for monitoring patients health; (xi) smartphone applications as diagnostic tools; (xii) AI-based clinician decision-making; (xiii) search engine for healthcare data flow; (xiv) data privacy and security; and (xiv) personalized treatments.

**Table 3:** Variability analysis of reviewed approaches.

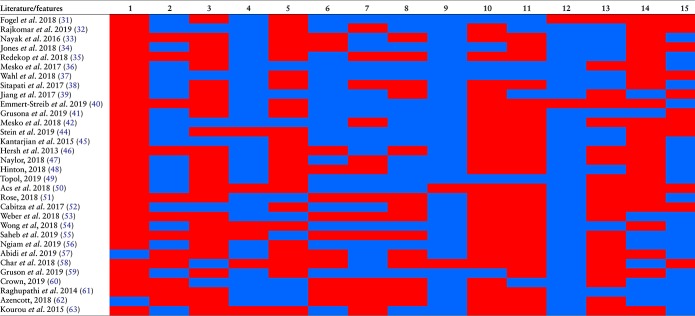

It is based on 15 different features, which includes 1: intelligent interface development; 2: next-gen radiology and imaging tools development; 3: global expansion of medical resources; 4: automated ETL, linkage and data mining in HER; 5: risk prediction and containment of antibiotics resistance; 6: pathology images analysis; 7: AI in machines and medical devices; 8: smart solutions and methods for cancer treatment; 9: EMR analysis for accurate risk predictors; 10: wearable devices for monitoring patients health; 11: smartphone applications as diagnostic tools; 12: AI-based clinician decision-making; 13: search engine for healthcare data flow; 14: data privacy and security; 15: personalized treatments. Color coding, red represents ‘absence’, and blue represents ‘presence’ of respective feature discussion

Although many AI and ML-based approaches have embraced the data gold rush in biomedicine, many concerns remain, which include integration of biomedical data located in heterogeneous data sources; handling extensively available irrelevant, error-prone and missing data; unnecessary follow-up diagnosis and treatment as a result of overloaded health information; loss of data privacy and reimagining of confidentiality; ethical strain of gaining industrial profits with clinical decision-support systems without informing the users; possibilities of patient data exploitation, employment insecurity due to governance and management of big data; regulation of healthcare data analysis according to principles of bioethical law; evidence-based observational data analysis and screening, slow data processing speed and difficulties in determining optimal parameters; difficulties in understanding structures of algorithms; possibility of too many overfitting attributes and its optimal network structure; handling continuous explanatory variables with more than two levels; difficulties in understanding odds and probabilities (119); correct predictor variable selection, reference group setup, and managing relationships between input and output variables (118,122); error-prone branching of missing values for an attribute (108); lack of data availability on social determinants of health; difficulties in handling redundant attributes, distribution of statistically independent attributes and management of class frequencies affecting accuracy (108); increased computational complexity with increasing number of attributes; computing probability of sequence observation; choosing accurate corresponding states and adjusting parameters (214); dealing with correlations between residues due to underlying decency assumption problem (215); and prediction modeling with biases in confounding, causal inference, complexity-based model selection, benchmark development and pragmatic interoperability including reproducibility and generalizability.

Potential solutions to these concerns are based on design and implementation of healthcare IT infrastructure (35,49); EMR documentation and automation of repetitive tasks with ML algorithms (31); deep learning to mine (41), train (39) and learn complex relationships between features and labels (32); ML algorithms to effectively link data between different platforms (33); development of effective clinical decision support systems (CDS) through the utilization of ML algorithms (38); translation of local languages into EHRs and cloud-based data sharing (37); deep learning for integrative EMR analysis from diverse sources (47); computer vision algorithms to identify accurate indigestion of medications for patient (31); deep learning for speech recognition, image interpretation and language translation (48); AI algorithms for diagnostic disease modeling and computer-aided design (CAD) (34); ML models to create knowledge base systems of phenotypes (40); ML algorithms to predict outbreak patterns and surveillance for new emergence (37); integrative approach to help decision-making processes between physicians and AI (89); ML algorithms to enhance and optimize cancer treatment and development of new drug treatments (45); ML algorithms to perform longitudinal population studies for analyzing the effects of treatments (56); ML-based hybrid model classifier to enhance overall healthcare predictability (72); and ML algorithms to transform clinical research into a much higher capacity and lower cost information processing care service (74).

We also considered most adaptable traditional (statistical) AI and ML algorithms (SVM, deep learning, logistic regression, discriminant analysis, decision tree, random forest, linear regression, naïve Bayes, KNN, HMM and genetic algorithm) in healthcare and justified their contributions with living examples for different clinical applications, e.g. deep neural network for diagnosing eye diseases (diabetic retinopathy) by analyzing retinal images; diagnosing cardiac anomalies by analyzing MRI images of heart ventricles; detecting malignant lung nodules by analyzing radiographs; grading prostate cancer tumors by analyzing ultrasound of biopsy cores; and producing glioma survival predictions by analyzing histological imaging and genomic marker data. Some other non-traditional algorithms were used, e.g., ‘Watson for Oncology’ (241,242) and ‘CURATE.AI’ (243,244), for analyzing EHR of cancer patients and recommend treatments and drug doses; TREWScore (245,246,247,248) for analyzing EHR for predicting septic shock risk and analyze CT brain scans for three-dimensional convolutional neural network neurological disorder classification; ‘PowerLook Density Assessment 3.4’ (249) for analyzing mammogram images for breast density assessment; ‘OsteoDetect’ (84,250,251) for detecting distal radius fractures in the wrist; cloud-based deep neural network algorithm for diagnosing cardiac anomalies and segmentation of lung and liver tumors by analyzing CT scans (56); and AI system for breast cancer prediction (252). Furthermore, major clinical oncological developments with the implementation of different AI and ML applications and algorithms include Chatbot; breast MRI interpretation; breast lesion classification; colorectal polyp classification; identification of colorectal cancer biomarkers in cell-free blood assays; gastric mucosal disease classification; detection of lung nodules in low-dose lung CT screening; prediction and evaluation of risk and malignancy and classification of dysplastic nevi, spitz nevi and basal and squamous cell carcinoma; detection of esophageal cancer; and identification of pancreatic cancer biomarkers (56).

The goal of this study is to highlight recent contributions and effectiveness of AI and ML in the development of computational systems towards better healthcare and precision medicine. We have reviewed and discussed multiple AI and ML-based approaches (Tables 1,2 and 3) and algorithms with variable colors. Deep learning (253,254) has proven to be one of the most trending algorithms today, but this does not undermine the importance of other machine learning algorithms. We believe that a right approach and algorithm should be chosen for the development of the most effective solutions to the targeted problems. In spite of various traditional and AI-based solutions, current limitations and challenges by the healthcare community include uneven distribution of resources towards the future of digital healthcare; business model unsustainability; disruption in the physician–patient relationships; hazards of dehumanization of healthcare data and job insecurity threat; prevention of early adopters for dragging down to the lowest common technology denominator; inconvenient adoption of digital processes for healthcare interoperability; shortages in development of customized IT infrastructure for data science; global unavailability of de-identified healthcare data for research; understanding of how healthcare data of different people relates to one another; need to harmonize big data with the definitions of the clinical phenotypes and diagnosis; limited EHR systems for assimilating operational and analytics interests; EHR offering limited patient participation; unavailability of high-quality open-source EHR systems inviting third-party extensions and assertions; and inflexible EHR database schemas not geared for precision medicine.

To effectively meet the goals of healthcare data analytics, while dealing with the aforementioned community, and traditional and AI-based challenges, significant efforts are required from experts in multidisciplinary sciences. To facilitate and improve public sector clinical research and practice, there is a critical need for academic frameworks that can connect operational and analytical systems in a way that experts from multiple domains can perform measurement and descriptive analysis, even without strong computational background. There is a need to develop standalone, user-friendly, standardized, open-source, and comprehensive solutions, which implement healthcare data analysis by connecting all kinds of patient data generated from any of the existing commercial EHR systems at any level, which includes patient’s demographic information, personal life style, medical history, recent visits to the practices, providers attended, diagnosis performed, lab tests conducted, longitudinal images, medications and procedures, samples taken for wet and dry lab experimentations for research and treatment of disease with no cure. Furthermore, the ideal system should be capable of automatically linking and communicating with similar systems.

Unmet clinical research and operational data analytics needs development of intelligent and secure systems to support practice transformation for implementing precision medicine at a global level. Overarching goals include new multi-functional platforms founded on the clinical, AI and scientific premise that integrates and analyzes heterogeneous clinical data received from multiple platforms. We need to implement and train AI classifiers at available structured clinical dataset of over a million subjects to help in early detection and diagnosis of common, frequently occurring and rare diseases and predicting the performance of provided treatments. We need to further explore AI methodologies to design models segregating disorders, identify causative medical conditions and determine the best drug therapies, especially when adjusted for age, race and gender. The goal should be to accelerate clinical care and discovery by satisfying research aims, improving quality and transition of care, obtaining actionable care gap-based information about patients and developing communication and coordination across hospitals, specialists, community-based providers, sub-acute care, nurses, quality inspectors, management, researchers and analysts. We need to strive to address possible challenges that continue to slow the advancements of this breakthrough treatment approach.

Along with AI- and ML-based methodological developments, it is important to address the issues related to healthcare data privacy and security. In most of the cases, academic and applied research environments do not have access to the healthcare data. The major reason is secure handling of protected health information (PHI) and lack of trust by the healthcare institutions in providing access to their medical records. We need to develop secure research-based HIPAA-compliant frameworks for efficient PHI storage, pre-processing, de-identification and integration to serve a large community of users, support organizational policies and provide efficient access and connectivity. It is mandatory to implement HIPAA rules for system user data security, which includes application and data criticality, risk management and analysis, information system activity review, contingency plan, device and media controls, disaster recovery plan, data backup plan, emergency mode operations plan, device and media controls, access controls, security incident procedures, vulnerability assessment, penetration testing, physical security, business associate agreements, polices and procedures, evaluation, audit, assignment of responsibility sanctions, workstation use and security. Furthermore, it is important to provide an external layer to the overall healthcare data analytics systems for placing clinical data in a distributed centralized structure; administering security by encrypting data as well as offering a multi-user-based graphical interface with controlled access; and supporting data backup with redundant data management plans.

## 8. Conclusion

Precision medicine is progressing but with many challenges lying ahead (255), which require addition of useful analytic tools, technologies, databases and approaches (4,6) to efficiently augment networking and interoperability of clinical, laboratory and public health systems, as well as address ethical and social issues related to the privacy and protection of healthcare and omics data with effective balance. This will also require more efficient management of massive amounts of generated data, as well as earlier mined consensus and actionable data. Most efforts involved currently are manual and time-consuming, whether it is extraction of healthcare data from operational clinical systems, identification of common and rare functional variants, metabolite penetrance using listed features and abnormalities, examining relations between genomic variations and metabolite levels, analyzing biochemical pathways in metabolites with patterns of multimodal distributions for candidate genes and management and assimilation of healthcare, along with epidemiological and omics data generated at each step of entry, production and analysis. Cutting-edge, new AI and ML-based big data platform development has the potential to revolutionize the field of medicine and improve the quality and transition of healthcare by intelligently analyzing structured clinical data available in great count and volume, posing unprecedented challenges in data storage, processing, exchange and curation, and developing a better understanding of biology.

## Supplementary Material

SupplementaryMaterial_Table_3_baaa010
